# Annotations

**Published:** 1911-09-16

**Authors:** 


### Annotations.

### The Local Government Board Report on Condensed Milks.

During such a summer as we have this year been enjoying, the milk question has become doubly acute, from the scarcity and from increased liability of the fluid to "turn" owing to the heat. Add to this the serious prevalence of gastro-enteritis among infants, and it may be imagined that the difficulties in connection with infant feeding have been considerably augmented, both for mothers and doctors. For these reasons the report just issued by the Local Government Board on condensed milks will be read with interest, though, in point of fact, it does not contain much that is new. As regards full-cream unsweetened condensed milk, it is allowed that under certain circumstances this may be a suitable substitute for cow's milk, but machine-skimmed condensed milk, of course, receives the severest condemnation. Some direct experiments carried out on milk artificially infected with tubercle bacilli and subsequently submitted to one of the ordinary trade processes for condensation, showed that the bacilli did not survive the process, and epidemiological evidence points to the conclusion that the germs of such diseases as scarlet fever, diphtheria, and enteric fever are destroyed in condensed milk. But other investigations showed that certain micro-organisms might survive the condensing process. The report is prefaced by a letter by Dr. Arthur Newsholme, in which the well-known dietetic disadvantages of any form of condensed milk are pointed out, and the necessity for administrative control of the sale of condensed milk for the food of infants is insisted upon; in particular, the labelling of all tins of skimmed milk as being unfit for the food of infants is urgently called for.

### The Release of Lunatics.

A painful case in the police courts, where a lady had to go to obtain protection from the impertinence of a lunatic, shows how difficult it is for anyone to say when a man who has once been mad may be regarded as sane. There are very few people who may not, under great mental or emotional stress, behave in an abnormal way, and in all classes there is a generous determination on the part of relatives to put up with such a spasmodic outburst. Very few people are sent to an asylum on the appearance of the first symptoms of insanity. It is usually when the lunatic begins to give trouble outside the home that he is sent to an asylum. Here he may appear to be perfectly sane—nay, he may be perfectly sane—the exciting cause of the brain disturbance being absent. But it does not follow that a patient who behaves like a sane person in the asylum will act like one elsewhere. This is known to all medical superintendents of asylums, and yet it is hardly possible for them to say, when friends ask for the patient's release, that he is insane. He is sane enough—under proper conditions. A society exists for paving the way between the asylum and the outer world by taking the released patient first to some quiet place, and letting the ordinary anxieties of the world reach him only by degrees. In many cases where family or financial troubles have caused the breakdown this plan is very useful. The mind is trained to its burden, and finally can carry it without undue strain. But in cases where there is one persistent obsession—as seems to have been the case with the man Craik, who assaulted Mrs. Egan-Newcomb—freedom means the opportunity for a repetition of the act which was already deemed to prove insanity. Such cases form a stumbling-block to asylum superintendents. The superintendent can only say that in the circumstances in which he knows him the patient is sane. He has no opportunity of testing him in those which first proved him mad. He may be doubtful of the wisdom of removal; but it is hard to keep a sane man cooped up among lunatics because there is the risk—a faint risk, it may be—of a relapse in another environment. He may warn the patient's friends to be careful and watch over him, but they, deceived by the obvious appearance of sanity, are apt to heed such warnings but little. They take their friend's sanity for granted, and thence come annoyances such as the one of which Mrs. Newcomb has complained, and oftentimes tragedies of a much more serious nature.

### A Crying Scandal.

A correspondent of the *Journal of Tropical Medicine and Hygiene* calls attention to a piece of official stupidity in Rhodesia which is, we trust, without a parallel anywhere else in the Empire, though unfortunately we have just as great an unremedied evil of a similar sort here at home. The writer of the letter has a son out in a malarious district of Rhodesia, to whom he lately sent a package of quinine. The recipient of the package was called upon to pay several shillings Customs duties, as quinine is included in the list of articles which pay an *ad valorem* duty on entering the country. It needs no emphasising that this is a veritable blood tax, a tax on human life and health, and one that in a tropical country there can be no shadow of palliation or excuse for. Surely it needs no medical training to realise that, and surely the exposure of this gruesome piece of official soullessness will inevitably and quickly lead to the placing of quinine on the free list. While we confidently hope that such a result will accrue from the publicity the matter has now received, it must be candidly admitted that as cruel and unjust and unscientific a piece of carelessness disfigures our own administration. So long as the Government is allowed to levy a duty on the rectified spirit used in the manufacture of chloroform and ether—which amounts practically to an *ad valorem* duty of 200 per cent. on these two anæsthetics—so long can we be reproached for the taxation of human suffering. Why should not both these disgraceful imposts be abolished together, that one in Rhodesia and this one at home?

